# Within-host competitive exclusion among species of the anther smut pathogen

**DOI:** 10.1186/1472-6785-9-11

**Published:** 2009-05-07

**Authors:** Alexander Gold, Tatiana Giraud, Michael E Hood

**Affiliations:** 1 Department of Biology, Amherst College, Amherst, MA 01002, USA; 2 CNRS, F-91405, Orsay cedex, France; 3 Ecologie, Systématique et Evolution, Centre National de la Recherche Scientifique; Université Paris-Sud, F-91405, Orsay cedex, France;

## Abstract

**Background:**

Host individuals represent an arena in which pathogens compete for resources and transmission opportunities, with major implications for the evolution of virulence and the structure of populations. Studies to date have focused on competitive interactions within pathogen species, and the level of antagonism tends to increase with the genetic distance between competitors. Anther-smut fungi, in the genus *Microbotryum*, have emerged as a tractable model for within-host competition. Here, using two pathogen species that are frequently found in sympatry, we investigated whether the antagonism seen among genotypes of the same species cascades up to influence competition among pathogen species.

**Results:**

Sequential inoculation of hosts showed that a resident infection most often excludes a challenging pathogen genotype, which is consistent with prior studies. However, the challenging pathogen was significantly more likely to invade the already-infected host if the resident infection was a conspecific genotype compared to challenges involving a closely related species. Moreover, when inter-specific co-infection occurred, the pathogens were highly segregated within the host, in contrast to intra-specific co-infection.

**Conclusion:**

We show evidence that competitive exclusion during infection can be greater among closely related pathogen species than among genotypes within species. This pattern follows from prior studies demonstrating that genetic distance and antagonistic interactions are positively correlated in *Microbotryum*. Fungal vegetative incompatibility is a likely mechanism of direct competitive interference, and has been shown in some fungi to be effective both within and across species boundaries. For systems where related pathogen species frequently co-occur in the same host populations, these competitive dynamics may substantially impact the spatial segregation of pathogen species.

## Background

Infection of a single host by multiple pathogen genotypes is a common phenomenon in a wide variety of diseases [1-4]. Theoretical studies focus heavily on virulence evolution and the consequence of whether the different pathogen genotypes remain together within the host, i.e. coinfection, or whether one competitively excludes the other, i.e. superinfection [5-9]. Some models postulate that genetic distance between pathogens determines cooperative versus competitive interactions within the individual host [10-12]. As pathogen genetic distance increases, there should be greater evolutionary conflict arising from sharing host resources, and thus enhanced selection for higher rates of exploitation associated with competition and virulence.

Only recently has a substantial number of empirical studies begun to describe within-host dynamics and help to ground the many theoretical models with examples from nature [2,13-15]. Pathogen genetic diversity has been shown to influence both the outcome of competition and whether a particular disease system assumes a coinfection or superinfection model. Research has however primarily focused on the intra-specific level of pathogen relatedness [11,13,16,17].

Closely related pathogen species may also co-occur on the same host due to host-shifts or speciation on a single host (e.g. in prior allopatry), thus creating opportunities for inter-specific competition. Examples include the frequent coinfection by *Plasmodium *species in regions of high malaria prevalence [3], which is associated with decreased virulence by *P. falciparum *when experiencing within-host competition against *P. vivax *[18,19]. Inter-specific competition has also been observed in the toad-polystome and the snail-trematode host-pathogen systems [20,21]. Despite such recent findings, however, it remains unclear for any particular study system whether antagonism seen at the intra-specific level extends in a similar manner to interactions among pathogen species. There are examples of the non-self recognition and antagonistic mechanisms fulfilling the same biological functions during intra- and inter-specific interactions, such as cuticular hydrocarbons in some insects [22] or the killing reactions of vegetative incompatibility in some filamentous fungi [23-25]. However, the extent to which such broadly effective antagonistic mechanisms are important to the evolution of complex disease systems remains largely unexplored.

An important model for within-host dynamics is the anther-smut disease, caused by fungi of the genus *Microbotryum *that infect plants in the Caryophyllaceae [1,16,17,26,27]. The fungus grows inside the meristematic regions of host plants and produces spores in the developing flowers, which are then transmitted to healthy plants by insect pollinators. Recent work has determined that mechanisms of competitive exclusion can result when the host is exposed to multiple pathogen genotypes [26] and, moreover, that the antagonism increases with genetic distance between pathogen genotypes of the same species [16]. The *Microbotryum *genotype that first infects the host most often excludes subsequent infections, and exclusion is more likely with distantly related conspecific genotypes. Within-host exclusion of less related genotypes in *Microbotryum *also has consequences for the spatial substructuring of pathogen populations, with naturally coinfected hosts containing pathogens that are more closely related than expected by chance alone [17].

Within the *Microbotryum *system, competition also exists between pathogen species and thus over substantially larger genetic distances than previously investigated with regard to within-host dynamics. Host-shifts are common [27-29], and some plant species are known to harbor multiple endemic species of *Microbotryum *[30,31]. The anther-smut disease of *Silene vulgaris *provides an excellent example, as several natural populations have been identified that contained sympatric mixtures of two pathogen species, *Microbotryum silenes-inflatae *and *Microbotryum lagerheimii *[30,31].

In the present study we characterize competitive interactions both within and between the species of *Microbotryum *found on *S. vulgaris *using sequential inoculations. By assessing whether the pathogen is more likely to colonize a host that is already infected by a member of the same or different *Microbotryum *species, this study sheds new light on whether the previously reported mechanisms of competition extend across species boundaries.

## Results

**Figure 1:**
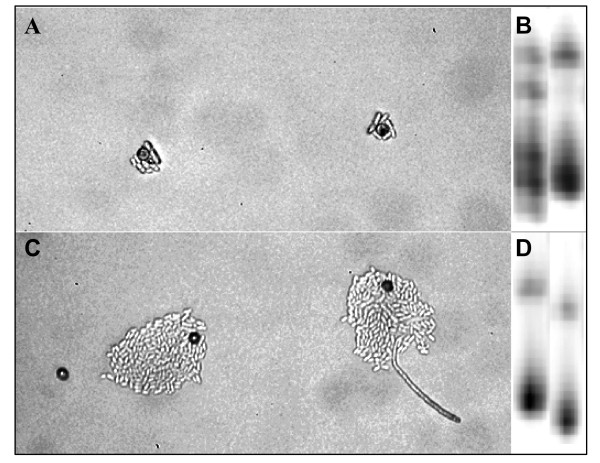
**Discrimination of *Microbotryum *genotypes used in experimental within-host competition**. Pathogen species were discriminated by colony morphology resulting from teliospore germination and growth on water agar after 72 hours at room temperature; *Microbotryum silenes-inflatae ***(A) **produced small colonies, while colonies of *Microbotryum lagerheimii ***(C) **contained many sporidia and often produced an infectious hypha. Within-species discrimination (**B **and **D**) used variation in PCR products using microsatellite primers (detailed in the Methods section).

Treatments that sequentially inoculated *S. vulgaris *plants with combinations of two pathogen genotypes from either of two *Microbotryum *species resulted in high rates of infection (Table 1). It is among these diseased plants that the outcome of within-host competition could be assessed by using morphological and genetic markers that discriminate each pathogen genotype used. Each control treatment (sequentially receiving a single inoculum type then water, or vice versa) resulted in infection rates of over 80%. Therefore, this source of host seeds was assumed susceptible to all pathogen genotypes at each of the inoculation time points. The frequency of disease across plants inoculated singly and repeatedly with the pathogen did not differ significantly (Wald's X^2 ^= 1.5, df = 1, P = 0.22). One of the four inter-specific competitions (*M. lagerheimii*-1 then *M. silenes-inflatae*-2), suffered severe mortality upon transfer from plate to soil, with one plant surviving to flower; this treatment was removed from further analysis as the mortality is believed to be due to technical issues during planting. The utility of morphological markers to differentiate *Microbotryum *species in inter-specific competitions was confirmed by assaying teliospore germination for all diseased plants of control treatments, which matched their expected growth morphologies as shown in Fig 1.

**Table 1:** Sequential Inoculation Treatments of *Microbotryum *species on *Silene vulgaris*.

		First Inoculation (Resident Infection)		
		*M. sil*-1	*M. lag*-1	Water
Second Inoculation (Challenge Infection)	*M. sil*-1		24 (27)	7 (7)
	*M. sil*-2	39 (39)	*	36 (36)
	*M. lag*-1	39 (39)		34 (35)
	*M. lag*-2	30 (31)	26 (31)	23 (29)
	Water	8 (8)	24 (30)	

Number of diseased plants per treatment, with total number of flowering plants in brackets. It was among the diseased plants that the pathogen genotype was determined. Columns represent the genotype infected first ("resident infection"), and rows represent the genotype infected second ("challenge infection"). "*M. sil" *= *Microbotryum silenes-inflatae*, "*M. lag*" = *Microbotryum lagerheimii*. Two distinct genotypes of each species were used ("1" or "2"), with the genotype used as the resident infection designated as "1" throughout. The treatment designated by * (*M. lagerheimii*-1 then *M. silenes-inflatae*-2) only had one plant survive to the flowering stage, and was dropped from subsequent analyses.

**Figure 2:**
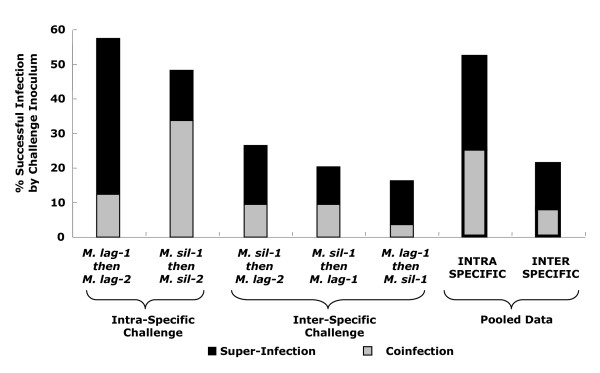
**Percent of plants per treatment expressing the challenger infection in at least one flower**. Total numbers of diseased plants per treatment are given in Table 1, among which the pathogen genotype causing disease was determined. Percentage bars are split into two sections, with grey representing coinfections (both genotypes expressed), and black representing super-infections (only the challenger expressed). "*M. sil*" = *Microbotryum silenes-inflatae*, "*M. lag*" = *Microbotryum lagerheimii*. The two genotypes used from each species are designated "1" and "2" after the species abbreviation. The remaining percentages of plants on the y-axis were those where only the resident inoculum was the detected.

The "resident" infection (i.e. the first of sequential inoculations) was the only pathogen genotype detected in the majority of plants for all treatments except the intra-specific treatment *M. lagerheimii*-1 then *M. lagerheimii*-2 (Fig. 2), as is consistent with previous results [26]. However, successful establishment of the "challenge" infection was detected significantly more often in intra-specific treatments than inter-specific treatments (Wald's X^2 ^= 16.3, df = 1, P < 0.001). The effect of genotype combinations within intra- and inter-specific treatments was not significant (Wald's X^2 ^= 1.3, df = 3, P = 0.72)

For each type of treatment, there was evidence of both coinfection and complete replacement of the resident pathogen by the challenge inoculum (i.e. super-infection). Whether establishment of the challenging inoculum was by coinfection versus replacement of the resident infection did not appear to differ between intra- and inter-specific treatments. The extremely high rates of disease among plants that were inoculated with one type of pathogen and then with water as a control (Table 1), particularly for the *M. silenes-inflatae *treatment, indicates that the super-infection category was most often the replacement of resident infection rather than failure of the first inoculation to colonize the host. Furthermore, an analysis was undertaken to assess coinfection of the same flowering stem, which requires persistence of multiple pathogen genotypes in a single host meristem, as compared to coinfection of separate stems originating from same plant rosette, differed among treatments. Results showed that significantly more of the intra-specific competitions expressed both pathogen genotypes on the same stem (10 of 16; average flowers and bolts sampled per plant were 5.1 and 2.2, respectively) as compared to the inter-specific competitions (0 of 8; average flowers and bolts sampled per plant were 5.5 and 2.9, respectively; Fisher's exact test, P = 0.040).

## Discussion

Success of the challenge inoculation during within-host competition by *Microbotryum *fungi was dependent on whether the pathogen interaction was between genotypes from the same or different species. The challenge inoculation was more successful at infecting a plant when the resident infection represented an intra-specific interaction, suggesting that the pathogen is more likely to share or concede its host to conspecific genotypes. When presented with a pathogen from another species, however, the resident infection more often excluded the challenger entirely, resulting in a lower rate of coinfection or replacement. These results substantially advance upon previous studies that investigated within-host competition between *Microbotryum *genotypes [16,17] by suggesting that competitive antagonism between coinfecting strains increases with genetic distance, not only at intra-specific levels of relatedness, but also to encompass between-species interactions.

Disease frequency of sequentially inoculated plants did not differ from singly inoculated plants, indicating that within-host competition between the pathogens did not have a protective effect on the host, as seen in some studies on other pathogens [11,18,32,33]. Also, it is important to note that the intra-specific competitors were not identical clones, in that they originated from different host populations and exhibited measurable genetic differences according to microsatellite variation.

These findings on within-host competition have important implications for disease ecology, particularly with the call for studies that move beyond the basic one-host one-pathogen framework [34]. The same mechanisms for non-self recognition by *Microbotryum *that were previously suggested to act during intra-specific competition [16,17,26] may be effective during competition between species as well. If so, the antagonistic continuum that spans species boundaries would be best understood by integrating population as well as community structures of the pathogens. These consequences are particularly significant for disease on *S. vulgaris*, where it has been shown that populations frequently contain two endemic species of *Microbotryum *[30,31]. In other host-pathogen combinations of the anther-smut disease, such as on the plants *Silene latifolia *and *Silene dioica*, sympatry of the hosts and their endemic diseases are common [35]. Host species in the caryophyllaceous genus *Dianthus *are also know to harbor multiple species of *Microbotryum *in the same geographic region [31,36], but the occurrence of mixed populations has not yet been investigated. Transient host shifts have been observed for the pathogen from *S. latifolia *onto sympatric *S. dioica *that are already maintaining infections by its host-specific *Microbotryum *lineage, and vice versa [35]. Therefore, a potential for strong exclusionary mechanisms by inter-specific pathogen competition to limit the persistence of incipient host shifts should be addressed by additional studies. Moreover, how selection on antagonistic mechanisms acting between pathogen species impacts the evolution of intra-specific competition, and vice versa, remains to be explored from theoretical and empirical grounds.

Exclusionary mechanisms during infection may also lead to different *Microbotryum *species achieving territorial segregation among host plants and contribute to barriers to gene flow between them. Pollinators would be less likely to transport a mixture of spores from different fungal species if they visit flowers consecutively on the same plant. Even when inter-specific coinfections were found, the two species exclusively colonized different flowering stems of the shared host. In this situation, both stems would have to produce infected flowers at the same time in order for pollinators to transmit the species together. In fact, the promotion of earlier flowering is one of the phenotypic effects of anther-smut disease upon its hosts [37], and the competition among pathogen genotypes is a likely selective force for manipulation of host phenology and may result in a degree of temporal as well as spatial segregation.

Given the previous findings that genetic distance between pathogens impacts the occurrence of multiple infections [16,17], it would be a simple extension to suggest that such competitive antagonism increases with genetic distance across species. Considering that the interactions are occurring *in planta*, however, it is difficult to investigate such mechanisms directly. Some potential mechanisms may be unlikely given our results. For example, disease resistance in *S. vulgaris *may be under control of a small number of genes [38] but there has been no evidence for a gene-for-gene resistance system [39] that could make hosts susceptible to one pathogen genotype over another. Where some plants have inducible resistances as the result of infection by fungi, such reactions are often non-specific in nature, and the difference between intra- and inter-specific challenges is more likely be due to other factors [40]. Moreover, the resistance of hosts used in this study was extremely low, and the dynamics of coinfection may be driven directly by pathogen-to-pathogen interactions.

Prior studies on *Microbotryum *have suggested that the fungal non-self recognition process of "vegetative incompatibility" likely serves as a mechanism of competitive exclusion [16,17]. Vegetative incompatibility is widespread amongst both ascomycete and basidiomycete fungi and is thought to have arisen to prevent the spread of infectious cytoplasmic elements or the sharing of resources [41]. The phenomenon is initiated when hyphae from unrelated individuals come into contact and respond to each other as distinct organisms, involving a cascade of gene expression and a killing reaction at the point of each hyphal connection [42,43]. Because vegetative incompatibility is governed by accumulated differences at a collection of *het *loci [42,43], more closely related individuals will have more similar alleles at these loci, potentially favoring their coexistence when colonizing in the same resource. In some fungi, vegetative incompatibility has been shown to affect interactions at both the intra- and inter-specific levels [23-25]. The observation that *Microbotryum *resides strictly within the microscopic corpus region of host meristems [44] has led to the suggestion that space is the primary limiting resource between competing genotypes [1,14,27]. In such confined regions, direct contact between coinfecting genotypes is likely and makes the vegetative incompatibility hypothesis more plausible. In addition, all cases of inter-specific coinfection resulted in the pathogen species segregating completely among different stems of the host plant, which indicates that the strongest antagonism disallowed coexistence in single meristems from which the separate flowers on a stem are produced.

## Conclusion

Here we have shown that the strength of competitive antagonism during infection by the anther smut fungi is correlated with the range of genetic distances that span from the intra-specific to the inter-specific levels. With the co-occurrence of multiple *Microbotryum *species on a single host, either due to separate endemic pathogen lineages or the high frequency of host shifts, the strength of competitive exclusion will have a major influence upon overall disease dynamics and potentially the extent of isolation between pathogen species. Further studies are needed to establish the cellular mechanisms at play during coinfection and to address the consequences of competition acting simultaneously at differently levels of selection.

## Methods

### Study System

The genus *Microbotryum *infecting the Caryophyllaceae comprises a complex of fungal species previously subsumed under the name *M. violaceum *(Pers.) Deml and Oberw. These basidiomycete pathogens reside in a highly restricted niche within the host, the corpus region of host meristems, and eventually produce diploid teliospores in the anther tissues of developing flowers [44]. Infected plants are usually completely sterilized because the disease also inhibits development of female structures [45]. Insect pollinators spread the teliospores to healthy plants, and upon germination the fungus undergoes meiosis. Conjugation between haploid cells of opposite mating types is a prerequisite for infection, with the mating system tending strongly toward selfing and automixis [46-48].

Recent phylogenetic studies have revealed the evolutionary independence of cryptic *Microbotryum *species on the Caryophyllaceae, and taxonomic revisions are currently in progress [31,49-53]. Two such species within the *Microbotryum *complex have been recognized for some time as causing disease in natural populations of the host *Silene vulgaris *[30,31]. Frequently found in sympatry on the same *S. vulgaris *host population [30,31], these two pathogen species are *Microbotryum lagerheimii *[49] and *Microbotryum silenes-inflatae*; referred to previously as MvSv1 and MvSv2, respectively [31]. This disease therefore provides an opportunity to study the dynamics of multiple infection and inter-specific competition without a confounding effect of the pathogens being specialized to different host species.

*Microbotryum *teliospores for this study were obtained during a 2006 census of *S. vulgaris *populations in Switzerland: specimens of *M. silenes-inflatae *were obtained from Davos (coordinates +46° 48' 47", +9° 49' 13") and St. Gotthard Pass (+46° 28' 36", +8° 26' 23"); specimens of *M. lagerheimii *were obtained from Oberalppass (+46° 37' 56", +8° 36' 33") and Bugnei (+46° 41' 1", +8° 47' 11"). Teliospores were collected from the field as the contents of mature infected flower buds. To ensure viable inoculum, teliospore germination was scored after incubation for 24 hours on potato dextrose agar at room temperature using a subjective scale of 1 to 5. A teliospore collection with the highest germination success (a score of 4 or 5) was chosen for use from each of the four populations of *Microbotryum*. Prior studies on *Microbotryum *from a variety of hosts, including from *S. vulgaris*, reveal that genetic variation within host-specific lineages is low [30]. *Microbotryum *species identification was obtained by DNA sequencing of the ribosomal internal transcribed spacer (ITS) and gamma-tubulin genes and comparing for identity with sequences from prior studies deposited in GenBank, NCBI [31,52].

Seeds of *S. vulgaris *were derived from a greenhouse collection of plants previously used to perform crosses between susceptible families [38]. The original host families were obtained from a population in Broadway, Rockingham County, Virginia, where *Microbotryum *naturally occurs on *S. vulgaris *as a result of a host shift from *Silene latifolia *[28,54].

### Sequential Inoculation

Seedlings of *S. vulgaris *were inoculated *in vitro *according to previously described methodology [26], with 7 days between sequential application of inoculum suspensions or water. Treatments are listed in Table 1, including the number of plants that became infected and could then be assessed for which pathogen genotype was the cause of disease. This interval between sequential inoculations was previously shown to allow the establishment of the first-inoculated genotype [26]. Briefly, seeds were surface sterilized and incubated on agar media until expansion of the cotyledons, at which time inoculum was applied to the apical meristem. The first inoculation was to establish the "resident" infections and consisted of 3 μl of a 1400 teliospores/μl suspension in water plus surfactant; the second inoculation was to present "challenge" infections and consisted of 3 μl of a 850 teliospores/μl suspension and was applied in the same manner to the apical meristem. Plants were transferred to soil and grown to maturity under greenhouse conditions in 115 cm^2 ^'Cone-tainers' (Stuewe and Sons, Inc., Corvallis, OR). The presence of disease was scored when the plants flowered by inspecting the anthers for teliospores. Each treatment was randomly assigned to 49 seedlings (resulting in a total of 588 plants treated), and plants were randomized for position in the greenhouse.

To avoid experimental contamination, mature flower buds were sampled the day before they would have opened, based upon the exertion of petals beyond the calyx teeth. The first mature flower bud produced by each plant was sampled, as well as subsequently produced buds on the same flowering stem and other stems originating from the plant rosette. Sampling was conducted over a period of 17 weeks.

When the competition was inter-specific, species assessment for the infections utilized differences in teliospore germination morphology after 72 hours of incubation on water agar at room temperature (Fig. 1A, C). When competition was intra-specific, pathogen genotypes were determined using variation in the microsatellites SVG8 and SVG5 resulting from PCR amplification with primers as described in past research [51] (Fig. 1B, D). DNA was extracted from infected anthers using the Chelex method [55]. Statistical comparisons were made using the generalized linear model procedures in SPSS version 12 (SPSS Inc., Chicago, Illinois, USA). A binomial logit function was assumed, with the test including the effect of intra- versus inter-specific treatments and the particular pathogen genotype combinations nested within the main effect. This procedure was also used to test whether there were different rates of infection among plants inoculated sequentially with the fungus versus receiving inoculum then water or vice versa. Due to the smaller sample size, a Fisher's exact test was used to assess whether co-infecting pathogen genotypes segregated differently among flowering stems depending upon intra- versus inter-specific treatments.

## Authors' contributions

AG and MEH designed and executed the experimental study, and TG provided the initial sources of inoculum and microsatellite genotyping of the experimental samples. AG drafted the manuscript, with revisions by MEH and TG. All authors read and approved the final manuscript.
